# NP-MRD: the Natural Products Magnetic Resonance Database

**DOI:** 10.1093/nar/gkab1052

**Published:** 2021-11-17

**Authors:** David S Wishart, Zinat Sayeeda, Zachary Budinski, AnChi Guo, Brian L Lee, Mark Berjanskii, Manoj Rout, Harrison Peters, Raynard Dizon, Robert Mah, Claudia Torres-Calzada, Mickel Hiebert-Giesbrecht, Dorna Varshavi, Dorsa Varshavi, Eponine Oler, Dana Allen, Xuan Cao, Vasuk Gautam, Andrew Maras, Ella F Poynton, Pegah Tavangar, Vera Yang, Jeffrey A van Santen, Rajarshi Ghosh, Saurav Sarma, Eleanor Knutson, Victoria Sullivan, Amy M Jystad, Ryan Renslow, Lloyd W Sumner, Roger G Linington, John R Cort

**Affiliations:** Department of Biological Sciences, University of Alberta, Edmonton, AB T6G 2E9, Canada; Department of Computing Science, University of Alberta, Edmonton, AB T6G 2E8, Canada; Department of Laboratory Medicine and Pathology, University of Alberta, Edmonton, AB T6G 2B7, Canada; Faculty of Pharmacy and Pharmaceutical Sciences, University of Alberta, Edmonton, AB T6G 2H7, Canada; Department of Computing Science, University of Alberta, Edmonton, AB T6G 2E8, Canada; Department of Biological Sciences, University of Alberta, Edmonton, AB T6G 2E9, Canada; Department of Biological Sciences, University of Alberta, Edmonton, AB T6G 2E9, Canada; Department of Biological Sciences, University of Alberta, Edmonton, AB T6G 2E9, Canada; Department of Biological Sciences, University of Alberta, Edmonton, AB T6G 2E9, Canada; Department of Biological Sciences, University of Alberta, Edmonton, AB T6G 2E9, Canada; Department of Biological Sciences, University of Alberta, Edmonton, AB T6G 2E9, Canada; Department of Biological Sciences, University of Alberta, Edmonton, AB T6G 2E9, Canada; Department of Biological Sciences, University of Alberta, Edmonton, AB T6G 2E9, Canada; Department of Biological Sciences, University of Alberta, Edmonton, AB T6G 2E9, Canada; Department of Biological Sciences, University of Alberta, Edmonton, AB T6G 2E9, Canada; Department of Biological Sciences, University of Alberta, Edmonton, AB T6G 2E9, Canada; Department of Biological Sciences, University of Alberta, Edmonton, AB T6G 2E9, Canada; Department of Biological Sciences, University of Alberta, Edmonton, AB T6G 2E9, Canada; Department of Biological Sciences, University of Alberta, Edmonton, AB T6G 2E9, Canada; Department of Biological Sciences, University of Alberta, Edmonton, AB T6G 2E9, Canada; Department of Biological Sciences, University of Alberta, Edmonton, AB T6G 2E9, Canada; Department of Chemistry, Simon Fraser University, Burnaby, BC V5A 1S6, Canada; Department of Chemistry, Simon Fraser University, Burnaby, BC V5A 1S6, Canada; Department of Chemistry, Simon Fraser University, Burnaby, BC V5A 1S6, Canada; Department of Chemistry, Simon Fraser University, Burnaby, BC V5A 1S6, Canada; Department of Chemistry, Simon Fraser University, Burnaby, BC V5A 1S6, Canada; Department of Biochemistry, University of Missouri, Columbia, MO 65211, USA; MU Metabolomics Center, University of Missouri, Columbia, MO 65211, USA; Bond Life Sciences Center, University of Missouri, Columbia, MO 65211, USA; Department of Biochemistry, University of Missouri, Columbia, MO 65211, USA; MU Metabolomics Center, University of Missouri, Columbia, MO 65211, USA; Bond Life Sciences Center, University of Missouri, Columbia, MO 65211, USA; Biological Sciences Division, Pacific Northwest National Laboratory, Richland, WA 99352, USA; Biological Sciences Division, Pacific Northwest National Laboratory, Richland, WA 99352, USA; Biological Sciences Division, Pacific Northwest National Laboratory, Richland, WA 99352, USA; Biological Sciences Division, Pacific Northwest National Laboratory, Richland, WA 99352, USA; Department of Biochemistry, University of Missouri, Columbia, MO 65211, USA; MU Metabolomics Center, University of Missouri, Columbia, MO 65211, USA; Bond Life Sciences Center, University of Missouri, Columbia, MO 65211, USA; Department of Chemistry, Simon Fraser University, Burnaby, BC V5A 1S6, Canada; Biological Sciences Division, Pacific Northwest National Laboratory, Richland, WA 99352, USA

## Abstract

The Natural Products Magnetic Resonance Database (NP-MRD) is a comprehensive, freely available electronic resource for the deposition, distribution, searching and retrieval of nuclear magnetic resonance (NMR) data on natural products, metabolites and other biologically derived chemicals. NMR spectroscopy has long been viewed as the ‘gold standard’ for the structure determination of novel natural products and novel metabolites. NMR is also widely used in natural product dereplication and the characterization of biofluid mixtures (metabolomics). All of these NMR applications require large collections of high quality, well-annotated, referential NMR spectra of pure compounds. Unfortunately, referential NMR spectral collections for natural products are quite limited. It is because of the critical need for dedicated, open access natural product NMR resources that the NP-MRD was funded by the National Institute of Health (NIH). Since its launch in 2020, the NP-MRD has grown quickly to become the world's largest repository for NMR data on natural products and other biological substances. It currently contains both structural and NMR data for nearly 41,000 natural product compounds from >7400 different living species. All structural, spectroscopic and descriptive data in the NP-MRD is interactively viewable, searchable and fully downloadable in multiple formats. Extensive hyperlinks to other databases of relevance are also provided. The NP-MRD also supports community deposition of NMR assignments and NMR spectra (1D and 2D) of natural products and related meta-data. The deposition system performs extensive data enrichment, automated data format conversion and spectral/assignment evaluation. Details of these database features, how they are implemented and plans for future upgrades are also provided. The NP-MRD is available at https://np-mrd.org.

## INTRODUCTION

Natural products are the bricks and mortar of biology, the foundation to biochemistry and the feedstock for medicinal chemistry. More than 66% of all drugs are derived from natural products (1), and nearly 98% of the compounds found in the human metabolome are natural products (2). Combined, the global natural product industry and the global pharmaceutical industry have an estimated market size of >$1.4 trillion/yr (3). In other words, natural products are not only essential for life, but are also essential to our quality of life. Strictly speaking natural products are small molecules (most are <2000 Da) that are fully or partially produced by living organisms. This includes any small molecule generated by bacteria, fungi, algae, plants, marine invertebrates, insects, fish or animals (including humans). While at least 400 000 natural products are known (4), it is estimated that there are >1 000 000 natural products that likely exist in living organisms (5). However, the true size of the natural product universe may well be several times larger.

Often natural products are divided into two categories: primary metabolites and secondary metabolites. A primary metabolite is a chemical compound that is essential to normal growth, development and reproduction, while a secondary metabolite is a non-essential metabolite that provides physiological benefits to its host and is present in a taxonomically restricted set of organisms or cells. Primary metabolites are of considerable interest to biochemists, biologists, metabolomics researchers and food chemists, while secondary metabolites are of greater interest to natural product chemists, organic synthetic chemists and medicinal chemists. However, as analytical tools grow in sensitivity and more is learned about their biological effects, secondary metabolites are gaining increasing attention in all fields of chemistry and biology.

The isolation and determination of natural product structures has occupied the attention of chemists for >200 years and has led (directly or indirectly) to the awarding of more than 30 Nobel prizes (https://www.nobelprize.org/prizes/lists/all-nobel-prizes/). Today, the standard method by which the absolute chemical structure is determined for all new and many previously identified natural products is by nuclear magnetic resonance (NMR) spectroscopy (6). NMR not only allows the determination of 3D molecular structures, it can also be used to determine the absolute configuration of chiral compounds and more commonly to determine the relative configuration of diastereomers (7). This information is vital for understanding the biological activity and biosynthetic origin of many natural products. In addition to its vital role in structure determination, NMR is also widely used in natural product dereplication (avoiding the re-identification of known natural products in extracts), in unknown identification (the characterization of previously unidentified natural products), in natural product purity assessment and in metabolomics (the characterization of metabolite extracts or mixtures) (8). These latter NMR applications require large and diverse collections of high quality, well-annotated, referential NMR spectra of pure natural products along with their associated structures and assignments (6–8). Unfortunately, these kinds of large reference NMR spectral collections are mostly unavailable or are very limited in scope.

Over the past two decades several ‘private’ databases have been developed to house NMR data for natural products. These include NAPROC-13 (9), which contains literature-derived ^13^C NMR spectral assignment data for over 21 000 natural products, CH-NMR-NP (https://www.j-resonance.com/en/nmrdb/), which contains literature-derived ^1^H/^13^C NMR assignment data for 29 500 natural products and Spektraris NMR (10) that contains about 2000 literature-derived NMR assignments for plant natural products. Other NMR databases also exist that ‘incidentally’ contain natural product NMR data. These include the spectral database of Japan or SBDS (https://sdbs.db.aist.go.jp), which houses ^1^H and ^13^C NMR data for over 900 natural products, the BioMagResBank or BMRB (11), which contains ^1^H and/or ^13^C NMR data for 2154 natural products/metabolites, the C6H6 repository (12), which includes ^1^H and ^13^C NMR data for 506 compounds, nmrshiftdb2 (13), which contains NMR data on nearly 1200 natural products along with spectral data on ∼43 000 other synthetic molecules and the Human Metabolome Database or HMDB (2) which contains ^1^H and ^13^C NMR data for 1480 natural products. Unlike NAPROC-13, CH-NMR-NP and Spektraris, these databases contain a significant number of experimental NMR spectra and NMR assignments.

While each of these NMR databases are excellent and well-maintained resources, none of them truly meets the needs of the natural products/metabolomics communities (14). Some are not web-accessible (Spektraris) or open access (NAPROC-13, SBDS), while others lack experimental NMR spectra (NAPROC-13, Spektraris, CH-NMR-NP). Most have very limited spectral search or comparison tools, many are too small or too limited in scope (HMDB, C6H6, BMRB), few have written descriptions or physicochemical information about the chemical compounds, most are not FAIR compliant (15), only three (C6H6, BMRB and HMDB) provide spectral data in standard data exchange formats, while just one (BMRB) is funded as a ‘permanent’ or persistent resource. More importantly, none of these databases have the capacity to accept external depositions from members of the natural product or metabolomics community.

Ideally, what is needed is an NMR natural product database that is sustainably funded, web-enabled, open access and FAIR compliant. Minimally, such a database should contain tens of thousands of experimentally acquired NMR spectra and NMR assignments for natural products, with the capacity to house NMR data on all known natural products. Housing experimental NMR data would ensure transparency, reproducibility and integrity in natural product research (14). Such a database should also provide rich, descriptive information about both the compounds and their corresponding NMR spectra/assignments. Furthermore, it should capture both biological taxonomic and chemotaxonomic information, it should be browsable, searchable and downloadable and it should support the use of standard NMR data exchange formats. In addition, it should provide objective, accessible measures of spectral or assignment quality/completeness and it should be able to accept external depositions (of NMR spectra and/or assignments) from the natural products or metabolomics communities, via direct deposition or via journal publishing agreements (14). Here we describe just such a database – The Natural Product Magnetic Resonance Database, or NP-MRD.

## DATABASE DESCRIPTION AND CONTENT

The NP-MRD has been funded by the National Institutes of Health (NIH), the National Center for Complimentary and Integrated Health (NCCIH) and Office of Dietary Supplements (ODS) to serve as the central clearing house for all NMR data generated by the natural products community. It has been designed to contain experimental NMR spectra (1D and 2D time-domain data as well as processed spectra), experimental ^1^H and ^13^C NMR chemical shift and J-coupling assignments, chemical structure data (2D and 3D structure data, atom numbering) and meta-data (nomenclature, literature sources, biological sources, taxonomic data, experimental methods, etc.) of known natural products. The NP-MRD is intended to be very inclusive and uses a very broad definition of what a natural product is. Any chemical compound (<2 kDa) produced or biologically transformed by bacteria, fungi, algae, plants, marine invertebrates, insects, fish or animals (including humans) is considered a natural product and can be included in the NP-MRD. The 2 kDa MW limit was chosen by the NP-MRD team to distinguish it from the BMRB and to allow NP-MRD’s interactive tools to operate with modest response delays. Larger biomolecules and biopolymers (>2 kDa) should be deposited in the BMRB.

The NP-MRD houses NMR data on primary metabolites, secondary metabolites and xenobiotic transformation products from all kingdoms of life. The NP-MRD also maintains a broad mandate regarding what kind of NMR data can be housed or deposited in the database. In particular, the NP-MRD actively seeks out, acquires and uploads legacy NMR data (spectra and assignments) of natural products derived from the literature, existing public databases and ‘private’ data archives. It also accepts new NMR data (spectra and assignments) submitted by depositors for novel natural products. In addition, the NP-MRD maintains a large collection of both predicted and simulated NMR data generated at multiple NMR spectrometer frequencies. These spectra are generated via traditional quantum mechanical spin simulation techniques (16), machine learning (17) and density functional theory (DFT) methods (18).

Recent advances in NMR theory along with continuing innovations in computing techniques are allowing remarkably accurate NMR spectral simulations and NMR parameter predictions to be made for many small molecules (16–19). In particular, it is now quite routine to generate accurate NMR spectra (which we call simulated NMR spectra) from published chemical shift assignments and known chemical structures. It is also possible to convert experimentally collected NMR spectra at one NMR frequency (say 600 MHz) and accurately simulate NMR spectra at multiple NMR frequencies (from 100 to 1000 MHz). Finally, it is also feasible to predict not only ^1^H and ^13^C shifts but also ^1^H and ^13^C NMR spectra from chemical structures with reasonably good accuracy. Therefore, including experimental, simulated and predicted NMR data in the NP-MRD ensures that the broadest possible coverage of natural products, NMR experiment types and NMR spectrometer frequencies is achieved. This is particularly important for dereplication efforts and for novel compound identification. It is also important for guiding/encouraging the natural products and metabolomics communities to acquire additional experimental NMR data to eventually replace and correct any predicted or simulated NMR data in the NP-MRD.

The NP-MRD is divided into two modules: (i) a database module and (ii) data deposition module (called NPN-Dep). The database module is the public-facing component of the NP-MRD and its layout, design and navigation will be discussed in the next section. NPN-Dep is for NMR data deposition into the NP-MRD by registered users and will be described in a later section. The current contents of the NP-MRD (Version 1.0) are listed in Table 1. As can be seen from this table, the NP-MRD has NMR data for >40 900 unique natural products or naturally occurring metabolites. More significantly, the NP-MRD has >817 000 NMR spectra (experimental, spin-simulated and predicted), making it far-and-away the largest NMR database for natural products in the world. Also, as shown in Table 1, the NMR data comes from six different sources, with most of the current data being ‘backfilled’ through accessing open-source, publicly available data from electronic databases such as the HMDB (2) and the BMRB (11). However, the NP-MRD is not simply a database mirror or a ‘database of databases’. Every entry in the NP-MRD is reformatted, corrected and converted to comply with the NP-MRD’s very high level of data standards. Each entry is also richly annotated with detailed information on compound names, synonyms, compound descriptions and structures. Every entry is associated with one or more annotated NMR spectra (measured, simulated and predicted), along with NMR assignments, biological sources (with taxonomic data), chemical taxonomy, measured and predicted physicochemical properties, database links and extensive literature references. NP-MRD also offers many high-end JavaScript molecular and spectral visualization tools and a wide variety of search, browse and comparison options that are not found in any existing NMR database. These value-added features, as well as many other still-to-be added features should make the NP-MRD a highly valuable addition to the field of natural product chemistry, medicinal chemistry and metabolomics.

**Table 1.** Current data content of NP-MRD (Version 1.0)

Source and type of data	Total
Total number of compounds in NP-MRD	40 908
Number of compounds from HMDB	879
Number of compounds from BMRB	284
Number of compounds from JEOL CH-NMR-NP	19 025
Number of compounds from NP Atlas	20 468
Number of compounds from NP-MRD Team Backfilling	185
Number of compounds deposited via NPN-Dep	279
Number of compounds with experimental NMR spectra	805
Number of compounds with experimental NMR assignments	19 840
Number of compounds with simulated NMR spectra	20 098
Number of compounds with pNMR spectra	20 700
Total number of experimental NMR spectra	1290
Number of experimental 1D NMR spectra	518
Number of experimental 2D NMR spectra	772
Number of experimental ^1^H NMR spectra	516
Number of experimental ^13^C NMR spectra	102
Total number of simulated NMR spectra	402 291
Number of simulated ^1^H NMR spectra	200 212
Number of simulated ^13^C NMR spectra	202 076
Total number of predicted NMR spectra	414 000
Number of predicted ^1^H NMR spectra	207 000
Number of predicted ^13^C NMR spectra	207 000
Total number of spectra (experimental, simulated and predicted)	817 681

## THE NP-MRD LAYOUT AND DATABASE NAVIGATION

**Figure 1.**
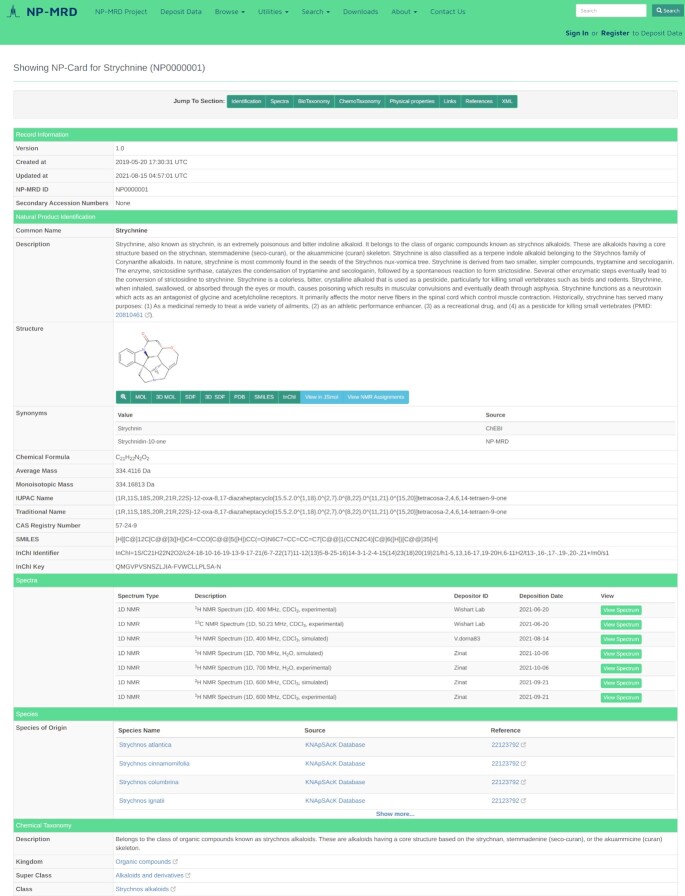
A screenshot and the NP-Card for the well-known plant-derived poison ‘strychnine’. This illustrates the rich annotations, the many data fields and the extensive viewing options available for nearly every compound in the NP-MRD.

The design of the NP-MRD and its user interface follows the same architecture and layout used for other well-known databases such as the HMDB (2) and DrugBank (20) that have been previously developed by our team. All chemical compounds in the NP-MRD along with their associated data are displayed in tables called ‘NP-Cards’ (i.e., natural product data cards). Each NP-Card is associated with a discrete natural product molecule and a unique natural product identifier (NP-MRD ID). The NP-Cards are similar in design to the MetaboCards used in the HMDB (2). Each NP-Card has multiple tabular data fields with labels on the left and data content on the right for facile display and navigation. An example NP-Card for the well-known toxic plant alkaloid known as ‘strychnine’ can be seen in Figure 1. As shown in this figure, each compound in the NP-MRD has an associated, unique 7-digit NP-MRD ID, a compound name, a detailed compound description (which is either hand-written or generated via a program called ChemoSummarizer (2)) and a thumbnail image of the structure. Larger versions of the chemical structure as well as links to MOL, 3D MOL, SDF, 3D SDF, PDB, SMILES and InChI files of that structure are available clicking the dark green tabs located below the thumbnail image. An interactive 3D JSMol (21) image of the molecule alone (with or without H atoms) as well as of the molecule with its NMR assignments is also accessible by clicking the blue tabs (‘View in JSMol’ and ‘View Assignments’) below the thumbnail image. Each NP-Card also provides information on known synonyms, the IUPAC name, the chemical formula, molecular weight(s), Chemical Abstract Service (CAS) Registry Number as well as information on the chemical taxonomy, the chemical substituents, the chemical ontology (as determined by ClassyFire (22)), and the biological source (with taxonomic information as provided by submitters or as extended by ChemoSummarizer). Additionally, information about experimentally measured or predicted physicochemical properties (melting/boiling points, solubility, log P, log S, etc.), hyperlinks to other well-known, online chemical databases (HMDB, ChEBI, PubChem, KEGG, etc.) and general references (with PubMed identifiers) are provided.

**Figure 2.**
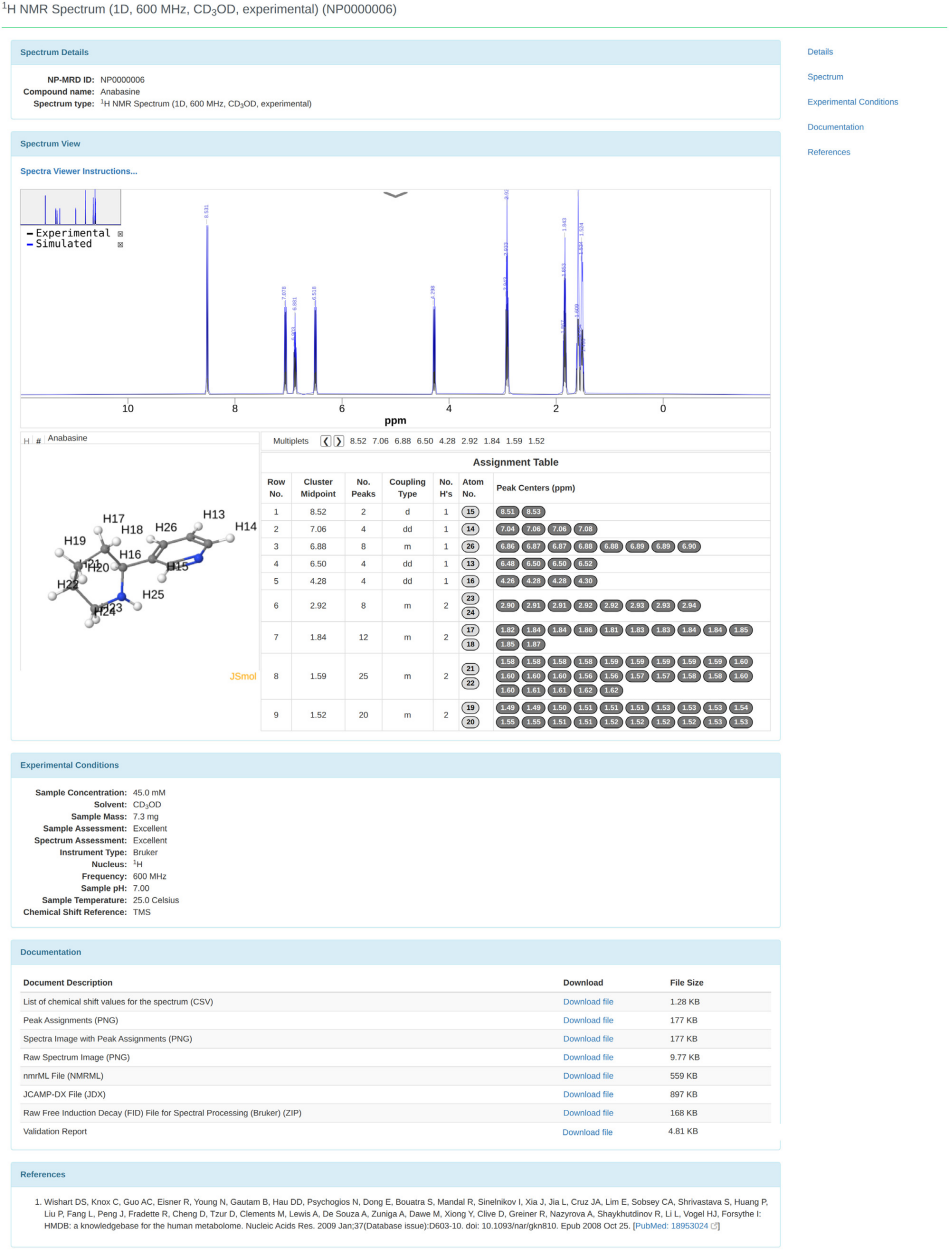
A screen shot of the NP-MRD spectral viewing page. This page displays all of the NMR data and associated meta-data, including general NMR spectral information, interactively viewable NMR spectra, experimental data, downloadable documentation and literature references.

Most importantly, each NP-Card provides detailed information and hyperlinks to the corresponding NMR spectral data (listed under the Spectra NP-Card header). This includes the spectrum type (1D, 2D, ^1^H/^13^C), a brief description of the NMR experiment, the deposition/depositor information, reference or citation information, hyperlinks to download the file(s) and a green ‘View Spectrum’ tab. Clicking on the ‘View Spectrum’ tab takes users to the NP-MRD spectral viewing page (Figure 2). This page displays all the NMR and associated metadata, including general NMR spectral information, interactively viewable NMR spectra, experimental data, downloadable documentation and literature references. This page also provides hyperlinks to navigate through the page(s). Both 1D and 2D NMR spectra can be viewed through this ‘View Spectrum’ page via a locally developed JavaScript spectral viewer called JSpectraViewer (23). JSpectraViewer or JSV displays NMR peak/chemical shift assignments both on the NMR spectrum and on the molecule itself, which is shown as a thumbnail image with numbered atoms and an assignment table. JSV also supports interactive spectral zooming, moving, gridding, scaling and image saving/downloading. The blue traces seen in 1D NMR spectra for JSV correspond to the predicted/simulated NMR spectra while the black traces correspond to the experimental NMR spectra. Only those entries with experimental NMR spectra will display both blue and black traces. Users can toggle between the black (experimentally acquired spectrum) and the blue (simulated/predicted spectra). Peak identification, spectral zooming and peak picking are also supported by the 2D version of JSV. Each NMR spectrum of a pure compound in the NP-MRD has downloadable information in the form of a set of peak lists (CSV format), peak assignments (CSV), spectral images (PNG), a spectral and/or assignment validation report and the actual or simulated NMR data in the form of nmrML (23) and JCAMP-DX files (24). If experimental data are available, the documentation section also provides native free-induction-decay (FID) or time-domain data in the original depositor format (Bruker, Varian, Agilent, JEOL).

Navigation through the NP-MRD is relatively simple. The NP-MRD home page has a green menu bar located at the top of the page with eight menu items listed, including **NP-MRD Project**, **Deposit Data**, **Browse**, **Utilities**, **Search**, **Downloads**, **About** and **Contact Us** (see Figure 1). The central portion of the NP-MRD home page also allows users to **Deposit Data** and **Browse Natural Products** through two slide-on hyperlinks or tabs. Under **NP-MRD Project**, users can learn about the project's background and the latest news regarding the NP-MRD. Under **Deposit Data**, users can use the NPN-Dep system to upload NMR data to the NP-MRD (described later). Under **Browse**, users can directly access the NP-MRD database and interactively browse several categories including ‘Natural Products’, ‘Chemical Classes’ or ‘Species of Origin’. Selecting ‘Natural Products’ produces a browsable, sortable table of all compounds in the NP-MRD. This table has six columns and 25 entries per page. Users can scroll through this table using a mouse or track pad or they may jump from page to page using the page navigation widget at the top and bottom of the **Browse** page. Users can also sort the table by NP-MRD ID, Name (alphabetically) or by mass. In addition, the NP-MRD **Browse** table can be filtered through a series of filter check boxes located at the top of the table. In particular, compound filtering or compound selection can be done by taxonomic kingdom of origin, NMR solvent type, NMR spectral quality, NMR spectral type and spectral nucleus. The NP-MRD filter tools provide a powerful route to select relevant subsections of the database for more efficient browsing or searching. Clicking on any NP-Card hyperlink (first column) or any compound name (second column) opens the NP-Card for the selected compound. Once the NP-Card is displayed, it can be easily navigated by scrolling through the page by jumping to specific sections (Identification, Spectra, BioTaxonomy, etc.) through green jump tabs located at the top of the page.

Under the **Browse** menu, users may also browse the NP-MRD via ‘Chemical Class’ or ‘Species of Origin’. Choosing ‘Chemical Class’ generates a browsable table with five columns and 30 entries per page. The five columns include Compound Name, Chemical Kingdom, Chemical Superclass, Chemical Class and Chemical Subclass, as determined by ClassyFire (22). Typing in the name of the compound or typing the name of the chemical class/kingdom at the top of each column allows one to sort or select compounds in the NP-MRD by specific chemical classes. Clicking on a specific chemical name launches the NP-Card for that compound, while clicking on the ‘Details’ tab displays the full chemical taxonomy for that compound. A list of available chemical taxonomies can be obtained by clicking the ‘tool-tip’ icon beside the corresponding column headers located at the top of the table.

Choosing ‘Species of Origin’ from the **Browse** pull-down produces a browsable table with five columns and 30 entries per page. The five columns include Species Name, Biological Kingdom, Biological Phylum, Biological Order and Biological Family, as determined by the NCBI Taxonomy listings (25). Entering in the name of a species or other taxonomic indicator at the top of each column allows one to find compounds in the NP-MRD belonging to specific taxa. The resulting table displays the NP-MRD ID, the compound name, its structure, the chemical formula and its taxonomic tree. A partial list of available Genus/Species, Kingdoms, Phyla, Orders and Families can be obtained by clicking the ‘tool-tip’ icon beside the corresponding column headers located at the top of the table. An autofill function suggests full taxonomic names as users enter their text in the taxonomy name boxes.

Under the **Utilities** menu item at the top of the NP-MRD home page, users may choose the ‘^1^H Chemical Shift Predictor’ or the ‘^13^C Chemical Shift Predictor’. To make the calculations sufficiently fast and accurate, both chemical shift predictors use a combination of machine learning techniques and HOSE-code methods that employ variations of the predictors available via NMRShiftDB (26). For both the ^1^H chemical shift predictor and the ^13^C chemical shift predictor, users must choose a solvent (the default is D_2_O, with options for CDCl_3_ and DMSO) and the chemical shift reference compound. To generate a chemical shift prediction, users must draw a structure of the compound of interest using the MarvinSketch applet and press the ‘Predict’ button. Structures may be drawn by pasting in a SMILES or an InChI string into the palette or users may draw the structure manually, one atom at a time, using the available MarvinSketch drawing tools. To test the system, users may press ‘Load Example’ to generate an example structure for performing a prediction. A typical prediction takes 3–5 seconds and the output displays a 3D structure of the molecule with the atom-specific numbering and a table of the predicted shifts with the corresponding atom numbers. The average chemical shift error is < 0.15 ppm for predicted ^1^H shifts and < 1.4 ppm for predicted ^13^C shifts (17,18,26).

Under the **Search** menu, users have the option of performing more sophisticated text searches with ‘Advanced Search’, simple text queries with ‘Text Query’, chemical structure searches with the ‘ChemQuery Structure Search’, a molecular weight query with ‘Molecular Weight Search’ and spectral searches with ‘NMR Search’. The Advanced Search allows users to search the database using a simplified, menu-driven SQL querying system that supports a wide range of conditions and predicates. Users can use menus to select different data fields within a given NP-Card (NP-MRD ID, name, description, molecular weight, chemical class, etc.) and apply various constraints or match conditions to one or more of these data fields. Users may also choose or alter what is displayed from these searches. An example search is provided by clicking the ‘Load Example’ button.

The ‘Text Query’ search allows simple text searches with single words, Boolean constraints (AND, OR) or with quotes to create searchable phrases. The same ‘Text Query’ search is also available through the ‘Search’ box located in the upper right of the NP-MRD menu bar. A typical query produces a browsable table of hits showing the compound name, the NP-MRD ID, the structure, the chemical formula, the molecular weight and the matching text with the word(s) highlighted in yellow.

The ‘ChemQuery Structure Search’ allows users to search the NP-MRD for structurally similar molecules. As before, the query structure must be uploaded using the MarvinSketch Applet. These structure searches may be filtered using a similarity threshold cutoff, a molecular weight cutoff, a search constraint or the maximum number of results. A typical structure query produces a browsable table showing the compound name, the NP-MRD ID, the structure, the chemical formula, the molecular weight and the Tanimoto score (to assess structural similarity). An example query structure is provided by clicking the ‘Load Example’ button.

The ‘Molecular Weight Search’ allows users to search the NP-MRD for molecules according to a molecular weight range (using either average molecular weight or monoisotopic molecular weight). A typical query produces a browsable table of hits showing the compound name, the NP-MRD ID, the structure, the chemical formula and the molecular weight. An example search is provided by clicking the ‘Load Example’ button.

The ‘NMR Search’ allows users to enter lists of ^1^H or ^13^C chemical shifts to search for spectral matches to experimental assignments and/or predicted assignments. Users must provide a chemical shift list, select the nucleus (^1^H or ^13^C) of interest and choose a chemical shift tolerance (default of 0.2 ppm for ^1^H and 2.0 ppm for ^13^C) before pressing the Search button. A typical query produces a browsable table showing the compound name, the NP-MRD ID, the structure, the chemical formula, the molecular weight, the chemical shift Dice score (a measure of chemical shift or spectral similarity), the fraction of peak matches and a colored tab that uses JSV to display a mirror plot of the query NMR spectrum matched against the matching NP-MRD NMR spectrum. An example is provided by clicking the ‘Load Example’ button. The NMR search is specifically designed to aid in novel compound identification, structure classification and dereplication.

Under the **Downloads** menu a full listing of all of the NP-MRD downloads are presented. All of the textual data (names, descriptions, chemical data) in the NP-MRD is available under ‘Natural Product Data’ in either XML or JSON format. All of the structural data in the NP-MRD is available under ‘Structures’ in SDF and SMILES format. Similarly, all of the NMR experimental data in the NP-MRD is available in native (Bruker, Varian, Agilent, JEOL time-domain format (FID)), while all peak list files for experimental, simulated and predicted NMR spectra are available in *.TXT format. Similarly, to support data exchange, all NMR spectral files for experimental simulated and predicted NMR are available as JCAMP-DX files and nmrML files. All downloadable files have a release date, a file size and a download link. The **About** section for NP-MRD contains summary information on the NP-MRD, its licensing structure and details about its adherence to FAIR database systems (15). It also contains up-to-date statistical information about the database, which is also largely summarized in Table 1.

## NP-MRD DATA DEPOSITION

The NP-MRD is not only mandated to serve as an open access natural product database, it is also required to accept and archive NMR data from the natural products and metabolomics communities. The NP-MRD data deposition system, which is called NPN-Dep (Natural Product NMR Deposition), is modeled after other public resource NMR data deposition systems such as NMRShiftDB (26) and BMRB (11). Currently, NPN-Dep, is designed to accept two types of external depositor data; (i) experimental NMR spectra (time-domain data) of pure natural products, with or without NMR assignments and (ii) NMR assignment data (^13^C or ^1^H) of pure natural products. As with other data deposition systems, the primary goal of NPN-Dep is to make data deposition into the NP-MRD, fast and easy, with a target deposition time of about 10 min per structure. The secondary goal is to provide a high level of quality control using a series of comprehensive validation steps to ensure accuracy and objectively assess the quality of deposited NMR assignments and NMR spectra. Other objectives for NPN-Dep are to ensure comprehensive, traceable data capture (to support scientific reproducibility and enhance data reliability) and to provide suitable enrichment and auto-annotation of all deposited data. These goals will ultimately allow both users and depositors to get more out of the database than what they put in.

To deposit data in the NP-MRD and to provide the necessary degree of data traceability, depositors must register with the database using an online form. This requires that depositors provide a valid email address which the requires user confirmation via email. Once registered, data depositors are taken to their own NPN-Dep home page, which allows them to launch their own data deposition process. Three deposition options are provided, two of which support online submissions and one which supports offline submissions. In particular, depositors may submit data for: (i) compounds with experimental FID (time domain) data and chemical shift assignments (online); (ii) compounds with chemical shift assignments only (online) and (iii) multiple compounds (with assignments) using the offline deposition option. Each depositor's NPN-Dep home page also tracks their past submissions with information on the NP-MRD ID, compound name, submission date, submission status and options to view/edit ongoing submissions.

**Figure 3.**
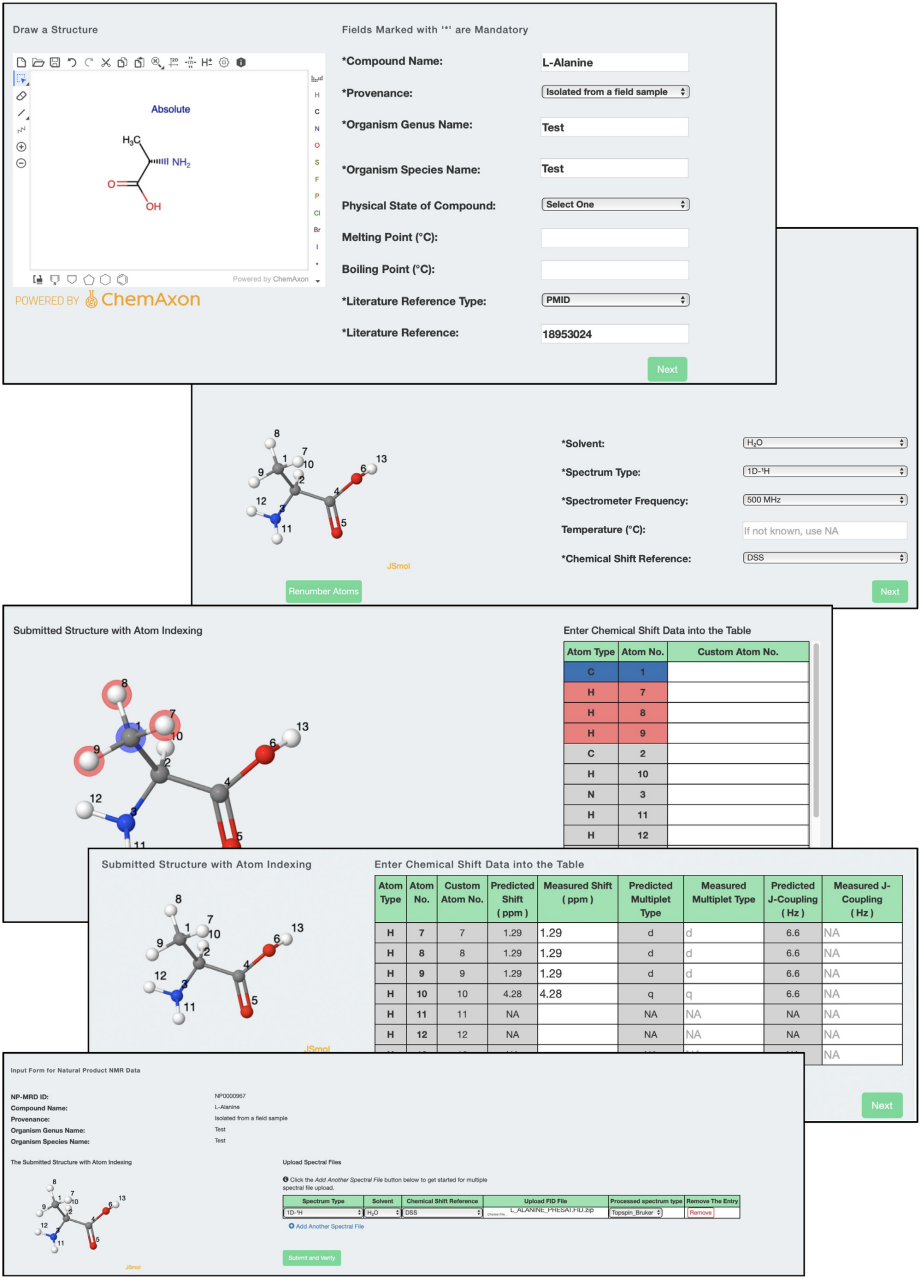
A screenshot montage showing the NPN-Dep data deposition process for the amino acid alanine.

For brevity, we will only describe the online data entry process. In particular, if Option 1 or Option 2 is selected, depositors are taken to the NPN-Dep home page which provides detailed multi-step instructions for the deposition process. A screenshot montage is shown in Figure 3 that outlines the data deposition process. In Step #1 of the deposition process, depositors must upload their compound structure into the MarvinSketch applet. A chemical structure is required by the program to check for identical or already existing entries in the NP-MRD. It is also used to generate appropriate atom numbering and to calculate NMR chemical shifts that will be used to help guide spectral assignment entry. In addition to uploading a structure, depositors are required to provide relevant meta-data about the compound such as the compound name, provenance (the source), physical state, melting/boiling point data and literature reference data. If the source information is unpublished or under review, the NP-MRD curation team will follow up with email queries to depositors to complete these fields. Once the required information in Step #1 is entered, and depositors press the ‘Next’ button, an automated check is performed to confirm if the structure is new to the NP-MRD. If a to-be-deposited structure already exists in the NP-MRD depositors are asked if the information they are uploading is new or different. If the structure is novel, or if the depositor has confirmed that an existing structure is associated with new/different information, depositors are taken to Step #2.

In Step #2, an interactive 3D structure of the molecule is displayed using JSMol with a default atom numbering scheme. Depositors have the option of re-numbering the atoms to create a more suitable atom numbering scheme using the ‘Renumber Atoms’ button. In addition to atom re-numbering, depositors must also enter additional NMR-related information about the NMR solvent, the spectrum type or NMR experiment (1D, 2D, homonuclear, etc.), the spectrometer frequency, the sample temperature and the chemical shift referencing compound. Once this information is completed, users can press the ‘Next’ button and are taken to Step #3 in the deposition process.

In Step #3, the structure with either the default or adjusted atom numbering is displayed via JSMol along with an editable Chemical Shift Data Table. This data table contains the atom types (C or H), the atom numbers, the predicted chemical shifts, the predicted multiplets and the predicted J couplings. The predicted chemical shifts, multiplet structures, and J-couplings are all generated using the chemical shift predictors and spectral simulators described earlier. In Step #3, depositors are required to enter their NMR assignments in the Chemical Shift Data Table using the labeled 3D structure as predicted/suggested NMR values as a guide. The intent of providing predicted NMR data is to ensure that depositors are entering their data correctly and to help reduce off-by-one errors that are common with online tabular data entry systems.

After their data is entered and the depositors press the ‘Save and Verify’ button, they are taken to Step #4 in the data submission process. This is the data and assignment verification step where depositors have the option of correcting or adding any missing data that has been flagged by NPN-Dep's automated data checkers. In particular, NP-MRD’s automated data checking utilities look for missing data or missing data fields, incompatible solvents/chemical shift references, unreasonable melting/boiling temperatures, incorrect or mis-spelled species names, unreasonable chemical shifts or unusual J-coupling constants. Depositors are asked to confirm if their data is correct or if errors are noticed, to make the necessary corrections. After any required changes or additions have been made, depositors are again asked to ‘Save and Verify’ their submission one more time. After the ‘Save and Verify’ button has been clicked the NPN-Dep system branches depending on whether Option 1 (spectra + assignments) or Option 2 (assignment only) had been selected at the beginning of the deposition process.

If Option 1 is selected, depositors are asked to upload their relevant NMR spectral files (i.e., the time-domain data). After uploading the data and pressing ‘Save and Verify’ they are presented with the processed NMR spectra through the JSpectraViewer applet for further inspection. During this step, NPN-Dep converts all deposited spectra and the submitted assignments to the appropriate nmrML formatted data (23) to facilitate their display by JSpectraViewer. This conversion is also done to support data downloads and encourage regular data exchange. nmrML is an XML mark-up language developed to encourage NMR data exchange (23). The nmrML data exchange format has been adopted by a number of large metabolomics databases as their NMR data exchange standard and has several programs that support the viewing, conversion and writing of nmrML file formats. NPN-Dep also converts uploaded spectral files to JCAMP-DX, an older or legacy NMR data exchange standard (23). Support for other exchange formats, including NMReDATA and NMRStar (11,12), is under development. If Option 2 is selected, NPN-Dep uses the submitted chemical structure and deposited NMR assignments to generate a series of simulated NMR spectra spanning 10 different NMR spectrometer frequencies (from 100 to 1000 MHz, in 100 MHz steps for ^1^H data and from 25 to 250 MHz in 25 MHz steps for ^13^C data). The resulting spectra are converted to nmrML formatted data so that they can be displayed via JSpectraViewer and so that the nmrML data files can be made available for download or data exchange.

In the final step of the data deposition process, NPN-Dep runs a series of data quality control checks using a tool called NPN-Validator. This validation step is modeled after a similar validation and evaluation process that is used by the Protein Data Bank for the validation of NMR protein data (27). For assignment-only data (Option 2) the NPN-Validator evaluates the quality of the assignments by determining if appropriate chemical shift reference standards have been used, enumerates how many chemical shifts assignments are missing, how many multiplet states are absent, how many *J*-coupling values are missing, and how many chemical shifts are out of range (3 standard deviations relative to predicted or expected shifts). NPN-Validator then scores the assignment quality relative to other deposited data sets in the NP-MRD and provides a color-graded scale (red for poor, blue for excellent) for each of the evaluated parameters. NPN-Validator also produces a relative score for the overall assignment quality.

For situations where assignments and spectra data are deposited, NPN-Validator runs not only an assignment assessment, but also a spectral quality assessment by looking at the quality of the NMR spectra, including the presence of chemical shift standards, the measured peak widths, the signal-to-noise ratio, the presence of solvent or contaminant peaks and the quality of phasing. The NPN spectral validator then scores the spectral quality relative to other deposited data sets in the NP-MRD. It also provides a color-graded scale (red for poor, blue for excellent) for each of the evaluated parameters as well as for the overall spectral quality. NPN-Validator's assignment and spectral validation reports are shown to depositors as part of the submission process and are included with the user-deposited data set (available with the NMR documentation files) when a deposited dataset is officially uploaded to the NP-MRD and made publicly available.

While depositors are completing their online submissions, the NP-MRD also conducts a series of automated data enrichment steps before the submission goes ‘live’ to the NP-MRD. This includes running ChemoSummarizer (2), DataWrangler (2) and ClassyFire (22) to identify relevant PubMed references, construct more detailed compound descriptions, classify the compound into specific chemical classes (chemotaxonomy), identify probable synonyms, calculate or predict various physical properties, identify known entries of the compound in other electronic databases, and determine or expand upon any taxonomic connections using the NCBI taxonomy system. These data enrichment tools have long been used to help annotate entries in the HMDB (2) and related databases. They are also particularly robust, fast, and fully traceable – thereby ensuring data integrity. It is only after these data enrichment steps are completed that a newly deposited NP-MRD entry is released publicly with an official NP-MRD ID and an official deposition date.

The online version of NPN-Dep has been operational since October 2020 and more than 270 NMR assignments and/or NMR spectra with assignments have been deposited by natural product or metabolomics community users to date. The offline version of NPN-Dep has been operational since June 2021.

## DATABASE IMPLEMENTATION

The NP-MRD was developed using Ruby, via Ruby on Rails, a development system that employs a concept called the Model-View-Controller (MVC). In the MVC framework, models respond and interact with the data by connecting to the database, views create the interface to show and interact with the data, and controllers connect the user to the views. This framework has allowed the NP-MRD programming team to rapidly develop, prototype and test all the NP-MRD modules and page views. All the data in the NP-MRD is stored in a MySQL relational database to facilitate rapid data extraction, tracking and storage. Hierarchical associations of records (‘trees’) are implemented as a nested set model inside a single database column of the tables. The raw information stored in the NP-MRD is dynamically extracted from the MySQL database and rendered into web pages by the HTML interface responder. Up to 1,000 of the most recent queries can be dynamically cached in memory for rapid content reloading. As with other databases developed in the Wishart laboratory (2,20), all chemical structures in the NP-MRD are hosted on a specially developed structure server called ‘Moldb’ and all NP-MRD spectral data are hosted on a server called ‘Specdb’. NP-MRD’s text search utilities are implemented using a locally developed ‘Unearth’ gem, which uses Elasticsearch indexing to allow rapid, flexible text searches. NP-MRD’s structure search utility uses ChemAxon's MarvinSketch molecular editor (implemented in JavaScript) coupled with ChemAxon's chemical similarity search algorithm. Other search utilities (spectral searches, Boolean text searches, mass or MW searches) are borrowed from a large collection of Ruby gems previously developed for the HMDB (2) and related databases (20, 28). The NP-MRD’s web interface has been built with the Bootstrap front-end framework while all the tables which correspond to different NP-MRD web pages are formatted using jQuery DataTables.

## DATA BACKFILLING, QUALITY CONTROL, CURATION, FAIR COMPLIANCE

As shown in Table 1, the NP-MRD consists of both user-deposited NMR data and curator back-filled data. The backfilled data currently represents the bulk of the data in the NP-MRD and is intended to help ‘stock the shelves’ of this newly launched database. It is our expectation that eventually the number of user-deposited entries will far exceed the number of backfilled data entries. Currently the backfilled data in the NP-MRD includes NMR data from: (i) other open-access NMR or metabolomics databases (HMDB, BMRB, CH-NMR-NP); (ii) NMR spectra/assignments specifically collected from journals and deposited by the NP-MRD team of curators and (iii) predicted ^1^H and ^13^C NMR spectra and assignments from selected structures deposited in the Natural Products Atlas (NP Atlas) collection of natural products (29). Most of the backfilled data collected from journals by the NP-MRD curation team has been uploaded through a specially developed, web-based curation tool called NPMRD_Curator. All the backfilled data have been extensively checked, updated, enriched and reformatted to comply with the NP-MRD data requirements and data exchange standards. The backfilling process for the NP-MRD has been done with the same quality assurance, quality control and data compilation procedures implemented for many of the databases developed by our team, including HMDB (2), DrugBank (20) and YMDB (28). In particular, all NMR data for the compounds from the HMDB, BMRB or CH-NMR-NP had to be of sufficiently high quality to comply with NP-MRD data standards. Molecules also had to fit with the accepted definition of a natural product (i.e. a small molecule that is fully or partially produced by living organisms). This was checked manually through an analysis of the species of origin and/or provided provenance data. Ambiguities were resolved through detailed literature searches. Because of these requirements (and because of redundancies), not all compounds nor all the NMR data in the various public NMR databases listed in Table 1 could be added to the NP-MRD. In order to ensure both completeness and correctness, each NP-MRD record that was manually or semi-automatically entered through the NPN-Dep system or the NPMRD_Curator system has also been manually reviewed and validated by a member of the curation team. Other members of the NP-MRD curation group routinely performed additional spot checks on the back-filled entries uploaded from the electronic databases.

To monitor the backfilling process, all the NP-MRD’s data is entered into a centralized, password-controlled database, allowing all changes and edits to NP-MRD to be monitored and time-stamped. Most senior members of the NP-MRD curation team have PhDs in chemistry, biochemistry or bioinformatics. Junior members of the curation team members are minimally required to be senior undergraduates or to have at least an undergraduate degree in bioinformatics or chemistry. This ensures that NP-MRD’s curators have sufficient chemical knowledge to understand and interpret the NMR and natural product literature. All NP-MRD curation team members were also given extensive training by the lead curator(s) in NMR spectral annotation and compound annotation via hands-on mentoring, text instructions, peer support, and tutorials.

Improvements and updates to NP-MRD’s content are an ongoing process and the data will be updated on a rolling basis. That is, minor corrections or small additions to an NP-MRD entry or to the layout will be done without a formal update announcement. However, significant changes, additions, or improvements to an individual NP-MRD entry will be listed in the NP-Card and the last update date will be modified to reflect any such changes. As this is only version 1.0 of NP-MRD, most entries have 2021 as the last update date. Large-scale updates and improvements to the database in the future will be given database version numbers (2.0, 3.0, etc.) and suitable database update dates. They will also be described in detail as publications or online update descriptions as appropriate.

As noted earlier, NP-MRD is FAIR compliant (15) and details regarding its ‘FAIRness’ are provided under the ‘About NP-MRD’ menu tab. To ensure findability, all entries in the NP-MRD have a unique and permanent 7-digit NP-MRD identifier. To ensure accessibility, the NP-MRD website is open and free and its data download operation is compatible with all modern web browsers. The NP-MRD’s downloadable spectral data is in the universally readable nmrML format. To ensure interoperability, all textual data and metadata in the NP-MRD are written in English, all spectral data are in the nmrML exchange format, all images are stored in PNG format, and all nomenclature for compounds and spectral data follows standard ontologies or vocabularies used to describe these entities. An extensive and well-annotated data download section is also provided with files available in standard CSV, JSON and XML formats. To ensure re-usability, all the data in the NP-MRD is extensively sourced with clear information on provenance. The data in the NP-MRD are available under a Creative Commons Attribution-NonCommercial 4.0 International License.

## LIMITATIONS AND FUTURE PLANS

It is important to remember that this is the first release of the NP-MRD and that the database is still evolving and maturing. As might be expected with any newly released database, there are going to be some obvious limitations and shortcomings. For instance, given that the natural product universe minimally contains >1 000 000 molecules and the current coverage of the NP-MRD is just 40,908 compounds, one obvious limitation is its relatively limited compound coverage. However, based on the current growth rate for the NP-MRD, we expect that by early 2022, that the NP-MRD will contain data for >400 000 molecules. We are also aware that many ‘classic’ natural products are still missing from the NP-MRD. This is because the NMR spectra for these compounds were either never collected/published or were collected/published in the 1950s or 1960s on very low field NMR instruments. Efforts are currently underway to acquire these compounds and collect their high field NMR spectra experimentally. If those compounds cannot be acquired, our intent is to predict their spectra using DFT and/or machine learning methods. We are also aware that more support of other NMR nuclei (^19^F, ^31^P, etc.), more support for the deposition of natural product mixtures and extracts and more extensive NMR search and spectral comparison functions need to be added. These functions and capabilities should come online in 2022. Likewise, the number of NMR utilities offered by the NP-MRD is still quite limited and efforts are underway to improve this situation. In particular, expanded spectral format conversion, spectral simulation, spectral processing (1D and 2D) and viewing, online DFT chemical shift calculation, mixture deconvolution, automated de-replication, computer-aided structure elucidation and spin matrix calculations are expected to be finished soon and should come online in 2022 and 2023. These additions are expected to make depositions to the NP-MRD much easier and the database itself more useful to the natural products community. In an effort to further increase user depositions to the database, the NP-MRD is currently in discussions with several journals and publishers to arrange for the deposition of published natural product NMR data to the NP-MRD as part of the standard publication process. This is expected to be in place in late 2022 and is projected to generate about 200 submissions a month.

The NP-MRD was established by the NIH to serve the natural products and metabolomics communities. As such the NP-MRD is always open to suggestions and ideas for improving this resource. We are particularly looking forward to receiving feedback for version 1.0 of NP-MRD, as this should make the team aware of other, less obvious shortcomings in the database's design, layout, logical flow and content. As always, we aim to be responsive to these comments and to engage the user community to make NP-MRD and its subsequent updates as useful, informative and reliable as possible.
